# A Simplified Plasticity Model Based on Synaptic Tagging and Capture Theory: Simplified STC

**DOI:** 10.3389/fncom.2021.798418

**Published:** 2022-02-11

**Authors:** Yiwen Ding, Ye Wang, Lihong Cao

**Affiliations:** ^1^State Key Laboratory of Media Convergence and Communication, Communication University of China, Beijing, China; ^2^Neuroscience and Intelligent Media Institute, Communication University of China, Beijing, China; ^3^State Key Laboratory of Mathematical Engineering and Advanced Computing, Wuxi, China

**Keywords:** synaptic plasticity, synaptic tagging and capture, calcium concentration, plasticity-related product (PRP), learning and memory

## Abstract

The formation and consolidation of memory play a vital role for survival in an ever-changing environment. In the brain, the change and stabilization of potentiated and depressed synapses are the neural basis of memory formation and maintenance. These changes can be induced by rather short stimuli (only a few seconds or even less) but should then be stable for months or years. Recently, the neural mechanism of conversion from rapid change during the early phase of synaptic plasticity into a stable memory trace in the late phase of synaptic plasticity is more and more clear at the protein and molecular levels, among which synaptic tagging and capture (STC) theory is one of the most popular theories. According to the STC theory, the change and stabilization of synaptic efficiency mainly depend on three processes related to calcium concentration, including synaptic tagging, synthesis of plasticity-related product (PRP), and the capture of PRP by tagged synapse. Based on the STC theory, several computational models are proposed. However, these models hardly take simplicity and biological interpretability into account simultaneously. Here, we propose a simplified STC (SM-STC) model to address this issue. In the SM-STC model, the concentration of calcium ion in each neuronal compartment and synapse is first calculated, and then the tag state of synapse and PRP are updated, and the coupling effect of tagged synapse and PRP is further considered to determine the plasticity state of the synapse, either potentiation or depression. We simulated the Schaffer collaterals pathway of the hippocampus targeting a multicompartment CA1 neuron for several hours of biological time. The results show that the SM-STC model can produce a broad range of experimental phenomena known in the physiological experiments, including long-term potentiation induced by high-frequency stimuli, long-term depression induced by low-frequency stimuli, and cross-capture with two stimuli separated by a delay. Thus, the SM-STC model proposed in this study provides an effective learning rule for brain-like computation on the premise of ensuring biological plausibility and computational efficiency.

## Introduction

The human brain contains billions of neurons connected to each other to form a complex neural network, and the connection between neurons is called the synapse. Specifically, the strength of synapses can be changed by perception and cognition processes, which is necessary and sufficient for the encoding and trace storage of memory (Takeuchi et al., 2014). The changes of synaptic strength (i.e., synaptic plasticity) can last for hours or even longer for memory maintenance. The persistent strengthening of synapses is termed long-term potentiation (LTP), and the reduction in the efficacy of neuronal synapses lasting hours or longer is called long-term depression (LTD). LTP and LTD could be triggered by a short duration of neural activity. Generally, high-frequency stimulus triggers LTP, whereas low-frequency stimulus triggers LTD (Bear and Malenka, 1994). As synaptic plasticity is essential for the development of the brain, especially for learning and memory (Martin et al., 2000; Takeuchi et al., 2014), how to accurately model synaptic plasticity is crucial for exploring the mechanisms under learning and memory and key for brain simulation.

In the past few decades, a variety of synaptic plasticity models represented by Hebbian rule (Hebb, 1949; Fusi, 2002), synaptic timing dependent plasticity (STDP) (Bi and Poo, 1998; Pfister and Gerstner, 2006), and the Bienenstock–Cooper–Munro (BCM) rule (Bienenstock et al., 1982; Shouval et al., 2002) have been proposed. However, they focus on the description of short-term plasticity and induction of early phase LTP and LTD; the maintenance (i.e., conversion from early to late-phase plasticity) that is critical for continual learning and memory consolidation was not under consideration.

A well-known theory of conversion from early to late-phase plasticity is synaptic tagging and capture (STC), which is supported by evidence both *in vitro* and *in vivo* (Frey and Morris, 1997; Redondo and Morris, 2011; Shires et al., 2012). The main hypothesis of STC theory is that long-term change of synaptic strength contains two necessary conditions. First, the dendritic spine on the postsynaptic neuron is activated by the presynaptic neuron and causes calcium influx into the spine, which makes the spine enters a tagged state (early phase plasticity, the calcium level determines whether the tagged state is potentiation or depression). The tagged state is a temporary structural state of the synapse that probably involves a large number of proteins and their interactions and could last for approximately 90 min (Redondo and Morris, 2011). Second, strong activation of a postsynaptic neuron causes the synthesis of plasticity-related product (PRP) in the soma or local dendritic domains. The molecular identity of all the PRPs is unknown but includes proteins such as Glur1, Homer1a, PKMζ, and ArC, and dendritic mRNAs as diffusible plasticity-related molecules; PRP could last for several hours (Redondo and Morris, 2011). When the tagged spine captures the PRP in the dendritic branch, the early phase plasticity would convert to late-phase plasticity. The tagging and capture processes exhibit symmetry, and therefore, PRP can be captured if they are synthesized either before or after the setting of the tag.

STC provides a new perspective for memory association and consolidation (Frey and Morris, 1998). First, STC greatly widens the time window of associative memory from short (less than 1 s) to long term (∼90 min), which enables events with a long time interval to be associated and helps memory integration. Second, a weak stimulus that tagged on the synapse could transform to long-term memory when it captures the PRP synthesized by strong stimuli, which might be the neural basis of the “flash memory” phenomenon. Third, due to the locality of PRP, capture preferentially occurs between stimulated spines that reside in the same dendritic branch, which affects the synaptic allocation in memory.

Computational models can bridge the gap between STC theory at the cellular level and memory at the behavior level. In recent years, a variety of plasticity models based on STC theory have been proposed. Clopath et al. (2008) propose TagTriC, which simulates the synaptic plasticity process of tagged synapse captures of PRP. TagTriC hypothesizes the formation of the tag, and PRP is associated with the membrane potential of neurons, which ignores the critical role of calcium concentration. Subsequently, Barrett et al. (2009) proposed a state transition model based on STC theory and simulated a broad range of experimental phenomena known as tagging experiments. In their model, the switch of synaptic states is considered as a Markov process; they use stimulus strength as the driving force and migrate the system to the next state with a certain probability. Smolen et al. (2012) propose a cascade model with the consideration of calcium concentration and a series of biochemical reactions on both tag and PRP, which simulated the biochemical mechanisms in the STC process precisely, but the model is too complex. Kastellakis et al. (2016) propose an STC model based on calcium concentration with relatively few equations and build a neural network containing hundreds of neurons to simulate the phenomenon that memories that occur closely are more likely to share neurons. In their model, the plasticity only occurs when both tag and PRP are present. Because early phase plasticity depends on tag other than PRP (Redondo and Morris, 2011), the early phase plasticity induced by tag cannot be simulated in their model, which limits its applications.

Therefore, the long-term plasticity models based on STC theory still have many shortcomings; it is necessary to propose a model that considers the biochemical mechanism under tagging and capture and is relatively easy to implement. To solve this issue, in this study, we propose a simplified long-term plasticity model based on STC theory: SM-STC. The SM-STC model considers the dynamic change of calcium concentration, its effects on tagged state and synthesis of PRP, and the coupling effects of tag and PRP on conversion from early to late-phase plasticity. In addition, the complexity and parameters in the model were largely reduced on the premise of ensuring biological plausibility. On this basis, we simulated a broad range of experimental phenomena known as tagging experiments. The proposed SM-STC model considers the biochemical mechanism and implement simplicity simultaneously, which may shed light on the learning and memory mechanism of the brain and facilitate the development of brain-like artificial intelligence.

## Materials and Methods

### Model Description

#### Presynaptic Plasticity

The SM-STC model considers both presynaptic and postsynaptic plasticity. The presynaptic plasticity is modulated by the neurotransmitter release amount and residual calcium level in the presynaptic neuron (Mongillo et al., 2008). A high neurotransmitter release amount and high residual calcium level induce synaptic facilitation, whereas a low neurotransmitter release amount and low residual calcium level induce synaptic depression. We define presynaptic efficiency *p* to describe the change of synapse induced by presynaptic activity, where *p* is multiplied with the synaptic conductance. The presynaptic efficiency *p* is defined as


(1) p=xu

(2) $$\frac{dx}{dt}=\frac{1-x}{\tau_{D}}-ux\delta \left(t-t_{sp}\right)$$

(3) $$\frac{du}{dt}=\frac{U-u}{\tau_{F}}+U\left(1-u\right)\delta \left(t-t_{sp}\right)$$

where *x* represents the normalized neurotransmitter release amount (0 < *x* < 1), *u* defines the residual calcium level, *U* is the baseline level of *u*, τ_*D*_ and τ_*F*_ are depression and facilitation time constants, δ is the Dirac delta function, and *t*_*sp*_ is the time of the presynaptic spike.

#### Postsynaptic Plasticity

**FIGURE 1:**
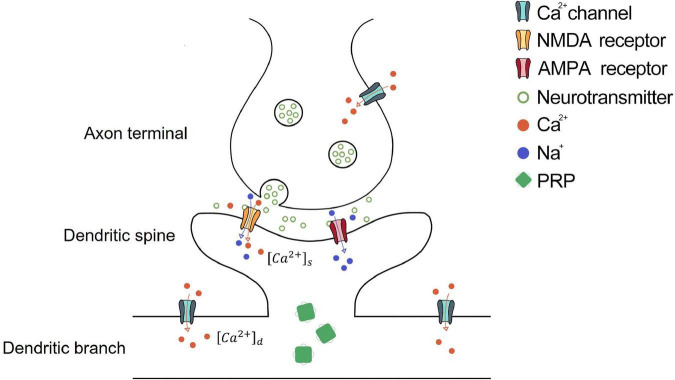
Schematic diagram of synaptic dynamics of STC theory. The activation of the presynaptic neuron induces calcium ion influx from the calcium channel and neurotransmitter release from the axon terminal. Then, the neurotransmitter binds the NMDA and AMPA receptors on the dendritic spine of the postsynaptic neuron. The activation of AMPA receptors causes an influx of sodium ions and cell membrane depolarization. NMDA receptors are voltage-gated receptors, which could be opened by neurotransmitter and cell membrane depolarization, causing the influx of sodium and calcium ions. The calcium ion influx through NMDA receptors enters the dendritic spine, which is [*Ca*^2 +^]_*s*_. When [*Ca*^2 +^]_*s*_ meets the condition of Eq. 5, the dendritic spine is tagged. The depolarized dendritic branch causes calcium influx through the calcium channel on the dendritic branch, which is [*Ca*^2 +^]_*d*_. When [*Ca*^2 +^]_*d*_ exceeds *Ca*0_*d*_, PRP begins synthesis. The potentiation or depression of the synapse occurs if the tagged dendritic spine captures PRP in the corresponding dendritic branch.

In the SM-STC model, the potentiation or depression induced by postsynaptic activity is determined by the tagged state of the dendritic spine and the PRP level of the corresponding dendritic branch. The tagged state of synapse *Tag* is related to the calcium ion concentration [*Ca*^2 +^]_*s*_ in the spine, the sign of *Tag* determines the direction of synaptic weight change, where positive *Tag* drives the synapse to potentiation, and negative *Tag* drives the synapse to depression, as shown in Figure 1. The evolution of *Tag* is defined, as


(4) $$\frac{d\left(Tag\right)}{dt}=-\alpha_{T}Tag+\beta_{T}\left(TagFlag-Tag\right)$$

where α_*T*_ is constant and *TagFlag* is an instantaneous variable and determined by the calcium concentration [*Ca*^2 +^]_*s*_ in the spine. If [*Ca*^2 +^]_*s*_ is below *Ca*0_*s*_, the synapse cannot be tagged, and the synaptic weight does not change; the *TagFlag* is set to 0. If [*Ca*^2 +^]_*s*_ is higher than *Ca*1_*s*_ (*Ca*1_*s*_ > *Ca*0_*s*_), then the synapse is tagged and enters an early phase LTP; *TagFlag* is set to 1. If [*Ca*^2 +^]_*s*_ is between *Ca*0_*s*_ and *Ca*1_*s*_, the synapse is tagged and enters an early phase LTD; *TagFlag* is set to -1 as follows:


(5) $$TagFlag=\left\{ \begin{array}{c} 0\\ -1\\ 1 \end{array}\right.\begin{array}{c} {\left[Ca^{2+}\right]}_{s}<Ca0_{s}\\ Ca0_{s}\leq {\left[Ca^{2+}\right]}_{s}\leq Ca1_{s}\\ {\left[Ca^{2+}\right]}_{s}>Ca1_{s} \end{array}$$

[*Ca*^2 +^]_*s*_ is modeled as the average calcium concentration through the NMDA receptor [*Ca*^2 +^]_*NMDA*_ within a time window of *t*_*Ca*_ as follows:


(6) $${\left[Ca^{2+}\right]}_{s}=\frac{1}{t_{Ca}}\sum {\left[Ca^{2+}\right]}_{NMDA}$$

where [*Ca*^2 +^]_*NMDA*_ is the concentration of calcium in the spine via NMDA receptor, and the process of calcium ion accumulation rather than transient effects is considered. β_*T*_ is a constant related to the state of synapse. β_*T*_ is defined as


(7) $$\beta_{T}=\{\begin{array}{c} 0\\ \beta_{T,LTD}\\ \beta_{T,LTP} \end{array}\begin{array}{c} if\;TagFlag=0\\ if\;TagFlag=-1\\ if\;TagFlag=1 \end{array}$$

where β_*T*, *LTD*_ and β_*T*, *LTP*_ are constant.

The synthesis of PRP is related to calcium concentration in the dendritic branch: [*Ca*^2 +^]_*d*_, as shown in Figure 1. [*Ca*^2 +^]_*d*_ is modeled as the average calcium concentration through the calcium channel [*Ca*^2 +^]_*channel*_ on the membrane within a time window of *t*_*Ca*_ as follows:


(8) $${\left[Ca^{2+}\right]}_{d}=\frac{1}{t_{Ca}}\sum {\left[Ca^{2+}\right]}_{channel}$$

When [*Ca*^2 +^]_*d*_ exceeds *Ca*0_*d*_, PRP begins synthesis. Due to the local effect of PRP (Govindarajan et al., 2011), we assume that PRP could not spread to other compartments. The change of *PRP* is modeled by a dual exponential function as follows:


(9) $$PRP=\sum_{i}\left(\exp\left(-\frac{t-t_{i}}{\tau_{r}}\right)-\exp\left(-\frac{t-t_{i}}{\tau_{d}}\right)\right)$$

where τ_*r*_ is the *PRP* rise time constant, τ_*d*_ is the *PRP* decay time constant, and *t*_*i*_ is the time when the calcium concentration in the dendrite meets the condition under which *PRP* can be synthesized (i.e., [*Ca*^2 +^]_*d*_≥*Ca*0_*d*_).

We define synaptic weight factor *z* to describe the change of synapse induced by postsynaptic activity, in which *z* is multiplied with the synaptic conductance. The synaptic weight factor *z* is determined as follows:


(10) $$z=\frac{\left(1-z_{l}\right)z_{h}e^{\mu y}+z_{l}\left(z_{h}-1\right)e^{-\mu y}}{\left(1-z_{l}\right)e^{\mu y}+\left(z_{h}-1\right)e^{-\mu y}}$$

(11) $$\frac{dy}{dt}=\left\{ \begin{array}{c} \frac{d\left(\gamma Tag\right)}{dt},ifPRP=0\\ \frac{Tag\cdot PRP}{\tau_{y}},ifPRP>0 \end{array}\right.$$

where *z*_*l*_ and *z*_*h*_ are the minimum and maximum weights that the synapse can achieve, and μ is the scale constant of *z*. *y* is an implicit variable; γ is scale constant of *y*. The calculation of *y* in early and late-phase plasticity are different. In early phase plasticity, the synapse enters a tagged state that is PRP synthesis independent (PRP = 0), and the change of *y* is only related to the tag. In late-phase plasticity, the change of *y* is proportional to the combined effects of tag and PRP (PRP>0) with a time constant of τ_*y*_.

Among the constants mentioned above, α_*T*_, τ_*y*_, β_*T*, *LTD*_, β_*T*, *LTP*_, τ_*r*_ and τ_*d*_ are time constant. Biologically, the dynamic change of tag and PRP takes time, so in our subsequent simulations, these time constants are set consistent with the biological time scale. Nevertheless, it is possible to adjust these time parameters to achieve accelerated learning. The values of the constant in both presynaptic and postsynaptic plasticity are shown in Table 1.

**TABLE 1:** Parameter values of SM-STC.

Symbol	Description	Value
*U*	Baseline level of *u*	0.2
τ_*D*_	Presynaptic depression time constant	0.2 s
τ_*F*_	Presynaptic facilitation time constant	1.5 s
α_*T*_	Tag related time constant	0.0007 s^–1^
β_*T,LTD*_	LTD-tag related time constant	0.2 s^–1^
β_*T,LTP*_	LTP-tag related time constant	1 s^–1^
*Ca*0_*s*_	LTD calcium threshold of spine	0.01 μmol/L
*Ca*1_*s*_	LTP calcium threshold of spine	0.2 μmol/L
*t_Ca_*	Calcium time window	0.1 s
τ_*r*_	*PRP* rise time constant	80 s
τ_*d*_	*PRP* decay time constant	9,000 s
*Ca*0_*d*_	Calcium threshold of dendrite	0.025 μmol/L
*z*_*l*_	Minimum weight the synapse can achieve	0.5
*z*_*h*_	Maximum weight the synapse can achieve	2
μ	Scale constant of z	0.1
γ	Scale constant of y	10
τ_*y*_	Time constant of y	30 s

### Simulation Experiments

**FIGURE 2:**
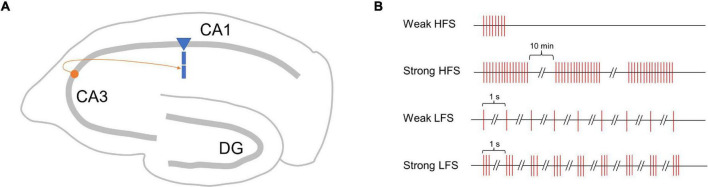
Schematic map of synapses simulated in this study and single-pathway stimulus patterns. **(A)** Synapse onto the CA1 pyramidal cell in the hippocampus via Schaffer collaterals. **(B)** Four stimulus patterns include weak HFS triggers early phase LTP (E-LTP), strong HFS triggers late-phase LTP (L-LTP), weak LFS triggers early phase LTD (E-LTD), and strong LFS triggers late-phase LTD (L-LTD) used in the single-pathway experiment.

To verify the performance of the proposed SM-STC model, we focus on the synapses onto the CA1 pyramidal cell in the hippocampus via Schaffer collaterals (Figure 2A), which is a typical plasticity system and has been well-studied in physiological experiments for synaptic plasticity (Dunwiddie and Lynch, 1978; Dudek and Bear, 1995). We designed two kinds of simulation experiments. The first one is a single-pathway experiment, which simulates early and late-phase plasticity by a single stimulus source (i.e., one presynaptic neuron). The second one is a two-pathway experiment, which simulates the conversion from early to late-phase plasticity as the PRP could be shared by different stimulus sources (i.e., two presynaptic neurons).

#### Neuronal and Synaptic Model

The neural activity of a CA1 pyramidal cell is simulated by a three-compartment ion-channel model (Wang et al., 2004), and the presynaptic neuron is simulated by Poisson spike trains. The synaptic connections between the presynaptic and the CA1 pyramidal cell contain AMPA and NMDA receptors. Changes in synaptic receptor conductance are modeled by the alpha function.

The calcium influx from NMDA receptors [*Ca*^2 +^]_*NMDA*_ is described as follows:


(12) $$\begin{array}{c} \frac{d\left({\left[Ca^{2+}\right]}_{NMDA}\right)}{dt}=\\ I_{NMDA}\left(t\right)-\left(\frac{1}{\tau_{Ca_{NMDA}}}\right){\left[Ca^{2+}\right]}_{NMDA} \end{array}$$

(13) $$\begin{array}{c} I_{NMDA}\left(t\right)=g_{NMDA}\left[\sum \frac{t-t_{s}}{\tau_{NMDA}}\exp\left(-\frac{t-t_{s}}{\tau_{NMDA}}\right)\right]\\ B\left(V\right)\left(V-E_{NMDA}\right) \end{array}$$

(14) $$B\left(V\right)=\frac{1}{1+\exp\left(-0.062V\right)\left(\frac{\left[Mg^{2+}\right]}{3.57}\right)}$$

where τ_*Ca*_*NMDA*__ is the time constant of calcium influx through the NMDA receptor, *I*_*NMDA*_ is the current via the NMDA receptor, *g*_*NMDA*_ is the conductance of the NMDA receptor, *E*_*NMDA*_ is the reversal potential of the NMDA receptor,τ_*NMDA*_ is the time constant of the NMDA receptor, *t*_*s*_ is the spike time of the presynaptic neuron, *V* is the membrane potential of the postsynaptic neuron, and [*Mg*^2 +^] is the magnesium ion constant.

Calcium influx from calcium channels on the dendritic branch [*Ca*^2 +^]_*channel*_ is described as follows:


(15) $$\frac{d\left({\left[Ca^{2+}\right]}_{channel}\right)}{dt}=-\alpha_{Ca}I_{Ca}\left(t\right)-\frac{{\left[Ca^{2+}\right]}_{channel}}{\tau_{Ca_{channel}}}$$

where α_*Ca*_ is the calcium correlated constant, *I*_*Ca*_ is the calcium ion current, and τ_*Ca*_*channel*__ is the time constant of the calcium channel.

#### Single-Pathway Experiment

Just as high-frequency stimuli (HFS) could induce LTP, low-frequency stimuli (LFS) could induce LTD; the stimuli strength matters whether early- or late-phase plasticity occurs (Sajikumar and Frey, 2004). Referring to physiological experiments (Frey and Morris, 1997, 1998), we designed four stimulus patterns with weak HFS triggers early phase LTP (E-LTP), strong HFS triggers late-phase LTP (L-LTP), weak LFS triggers early phase LTD (E-LTD), and strong LFS triggers late-phase LTD (L-LTD), as shown in Figure 2B. The weak HFS contains a single tetanus (100 Hz), and the stimulus sustains for 0.2 s. The strong HFS contains three times of tetanus (100 Hz), each sustains for 1 s, separated by 10 min intervals. The weak LFS contains 900 times 1 Hz stimulus, and each stimulus lasts for 1 s, that is, the next stimulus starts 1 s after the onset of the previous stimulus. The strong LFS contains 900 bursts of three stimuli at 20 Hz, and the next stimuli start 1 s after the onset of the previous stimuli. For each stimulus pattern, a total of 300 min of biological time was simulated. Each experiment was run 10 times with different random seeds to generate Poisson spike trains of the presynaptic neuron.

#### Two-Pathway Experiment

The two-pathway experiment focuses on the phenomenon of a weak E-LTP/D-inducing protocol delivered to one pathway rescued into an L-LTP/D if a strong L-LTP/D-inducing protocol is delivered to the other pathway at around the same time. This phenomenon is known as cross-capture (Sajikumar et al., 2005; Reymann and Frey, 2007), with which the tag caused by one pathway captures PRP that is synthesized by another pathway. Moreover, the phenomenon is reciprocal as a rescue of E-LTD into L-LTD occurs when another pathway experiences a strong L-LTP/D-inducing protocol.

Due to the cross-capture phenomenon and four types of stimulus pattern in each pathway, there are 16 combinations in two-pathway experiments. If the stimulus in both pathways is strong, then each pathway could synthesize PRP by itself and does not need to capture PRP synthesized by another pathway. If the stimulus in both pathways is weak, then neither stimulus could trigger PRP synthesis, and none of them could convert early phase LTP/D to late phase LTP/D. Thus, there are eight combinations left, as shown in Table 2. The weak HFS, strong HFS, weak LFS, and strong LFS used in the two-pathway experiment are the same as the one-pathway experiment. The time interval between the two pathway stimuli is 30 min; that is, the stimuli from the second pathway starts 30 min after the stimuli of the first pathway. A total of 300 min of biological time was simulated for each stimulus pattern. Each experiment was run 10 times with different random seeds to generate Poisson spike trains of the presynaptic neuron.

**TABLE 2:** Stimuli combinations in the two-pathway experiment.

		Stimuli in the second pathway			
		Strong HFS	Weak HFS	Strong LFS	Weak LFS
Stimuli in the first pathway	Strong HFS	X	√	X	√
	Weak HFS	√	X	√	X
	Strong LFS	X	√	X	√
	Weak LFS	√	X	√	X

## Results

### Single-Pathway Experiment

#### Weak High-Frequency Stimuli Induce Early Phase LTP

**FIGURE 3:**
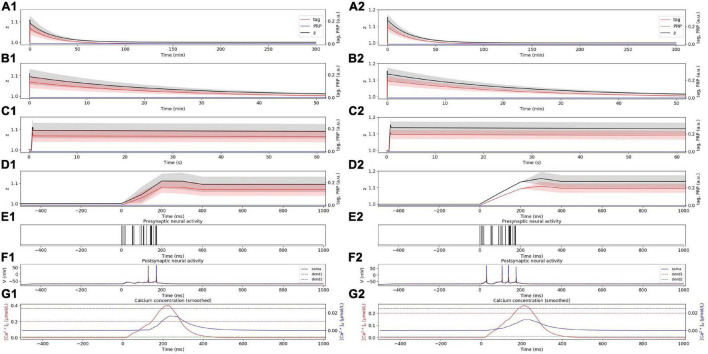
E-LTP induced by weak HFS. **(A)** Dynamic change of tag (red), PRP (blue), and z (black) during the whole simulation time (300 min) without **(A1)** and with **(A2)** considering presynaptic plasticity. **(B)** Dynamic change of tag (red), PRP (blue), and z (black) during the first 50 min without **(B1)** and with **(B2)** considering presynaptic plasticity. **(C)** Dynamic change of tag (red), PRP (blue) and z (black) during the first 60 s without **(C1)** and with **(C2)** considering presynaptic plasticity. **(D)** Dynamic change of tag (red), PRP (blue) and z (black) during the first 1 s without **(D1)** and with **(D2)** considering presynaptic plasticity. **(E)** Activity of presynaptic neuron during the first tetanus stimulus without **(E1)** and with **(E2)** considering presynaptic plasticity. **(F)** Activity of postsynaptic CA1 neuron during the first tetanus stimulus without **(F1)** and with **(F2)** considering presynaptic plasticity, the membrane potential of soma, dendrite 1, and dendrite 2 are shown in blue, red, and black. **(G)** Calcium concentration in spine (red) and dendritic branch (blue) during the first tetanus stimulus without **(G1)** and with **(G2)** considering presynaptic plasticity, calcium threshold for tag and PRP are shown in green (*Ca*0_*s*_), red (*Ca*1_*s*_), and blue (*Ca*0_*d*_) dotted lines. The PRP has been magnified 200 times for clearer display, and the shaded areas stand for standard deviation.

We first compared the consistency between the simulation results of SM-STC and physiological experiments. The physiological experiments supporting STC theory are *in vitro*, that is, the neurons are stimulated with different frequencies without presynaptic plasticity. Therefore, in the following simulation, we do not consider presynaptic plasticity, but only study the role of postsynaptic plasticity. In the weak HFS experiment, the dynamic changes of tag, PRP, and z under weak HFS during the whole simulation time are calculated, as shown in Figure 3A1. The stimulation at the initial time caused the changes of tag; thus, we zoom in to visualize the changes of tag, PRP, and z in the first 50 min (Figure 3B1), 60 s (Figure 3C1), and 1 s (Figure 3D1). To visualize the neural activity and calcium concentration behind tag and PRP synthesis, the presynaptic neural activity, postsynaptic neural activity, and calcium concentration in both spine and dendritic branch during the first tetanus stimulus are shown in Figures 3E1,F1,G1. The calcium concentration (Figure 3G1) in the spine is higher than *Ca*1_*s*_ (red dotted line), which is high enough to induce the LTP tag and further results in E-LTP. However, the stimulus is too weak to induce adequate calcium concentration in the dendritic branch (lower than *Ca*0_*d*_, blue dotted line), which results in no PRP synthesis, and the E-LTP could not convert to L-LTP. The synaptic potentiation lasts for about 90 min, and the trend of synaptic weight is consistent with observations in biophysical experiments (Figure 4A of Frey and Morris, 1997).

Next, we are curious about the influence of presynaptic and postsynaptic plasticity on the synaptic connection. Therefore, we consider presynaptic plasticity in section “Presynaptic Plasticity” and repeat the simulation experiment above. The results show that the tendency of tag, PRP, and z does not change after the addition of presynaptic plasticity, and we found the amplitude increase of E-LTP (larger z), as shown in Figures 3A2–3G2.

#### Weak Low-Frequency Stimuli Induce Early Phase LTD

**FIGURE 4:**
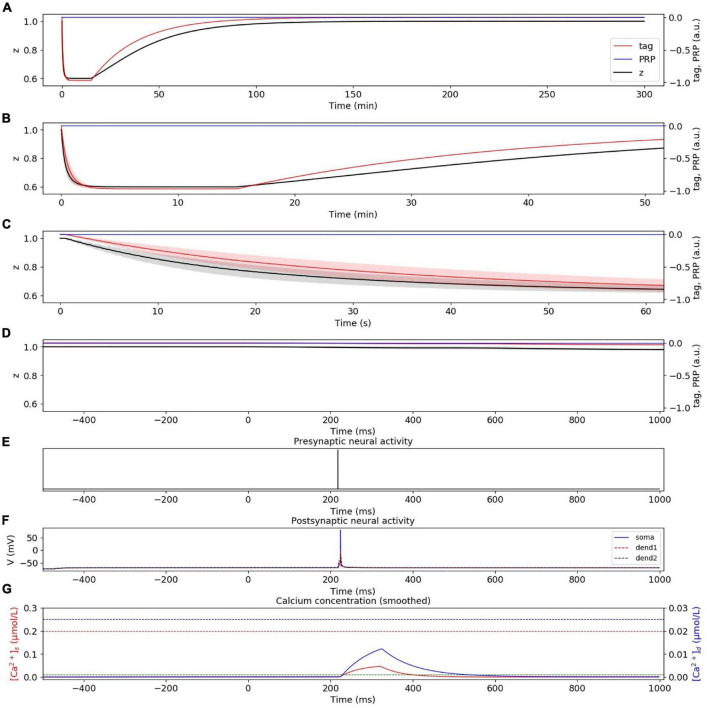
E-LTD induced by weak LFS without considering presynaptic plasticity. **(A)** Dynamic change of tag (red), PRP (blue), and z (black) during the whole simulation time (300 min). **(B)** Dynamic change of tag (red), PRP (blue), and z (black) during the first 50 min. **(C)** Dynamic change of tag (red), PRP (blue), and z (black) during the first 60 s. **(D)** Dynamic change of tag (red), PRP (blue), and z (black) during the first 1 s. **(E)** Activity of presynaptic neuron during the first 1 Hz stimulus. **(F)** Activity of postsynaptic CA1 neuron; the membrane potential of soma, dendrite 1, and dendrite 2 are shown in blue, red, and black. **(G)** Calcium concentration in the spine (red) and dendritic branch (blue); calcium threshold for tag and PRP are shown in green (*Ca*0_*s*_), red (*Ca*1_*s*_), and blue (*Ca*0_*d*_) dotted lines. The PRP has been magnified 200 times for clearer display, and the shaded areas stand for standard deviation.

In the weak LFS experiment without considering presynaptic plasticity, the dynamic changes of tag, PRP, and z under weak LFS are calculated, as shown in Figure 4A. The stimulation at the initial time caused the changes of tag; thus, we zoom in to visualize the changes of tag, PRP, and z in the first 50 min (Figure 4B), 60 s (Figure 4C), and 1 s (Figure 4D). To visualize the neural activity and calcium concentration behind tag and PRP synthesis, the presynaptic neural activity, postsynaptic neural activity, and calcium concentration in both spine and dendritic branch during the first 1 Hz stimulus are shown in Figures 4E–G. The calcium concentration (Figure 4G) in the spine is between *Ca*0_*s*_ (green dotted line) and *Ca*1_*s*_ (red dotted line), which induces LTD tag and further results in E-LTD. However, the stimulus is too weak to induce adequate calcium concentration in the dendritic branch (lower than *Ca*0_*d*_, blue dotted line), which results in no PRP synthesis, and the E-LTD could not convert to L-LTD. The synaptic depression lasts for about 90 min, and the dynamic change of synaptic weight is consistent with recordings in the physiological experiment (Figure 2A of Sajikumar and Frey, 2004). Moreover, the addition of presynaptic plasticity did not change the tendency of tag, PRP, and z, as shown in Supplementary Figure 1.

#### Strong High-Frequency Stimuli Induce Late-Phase LTP

**FIGURE 5:**
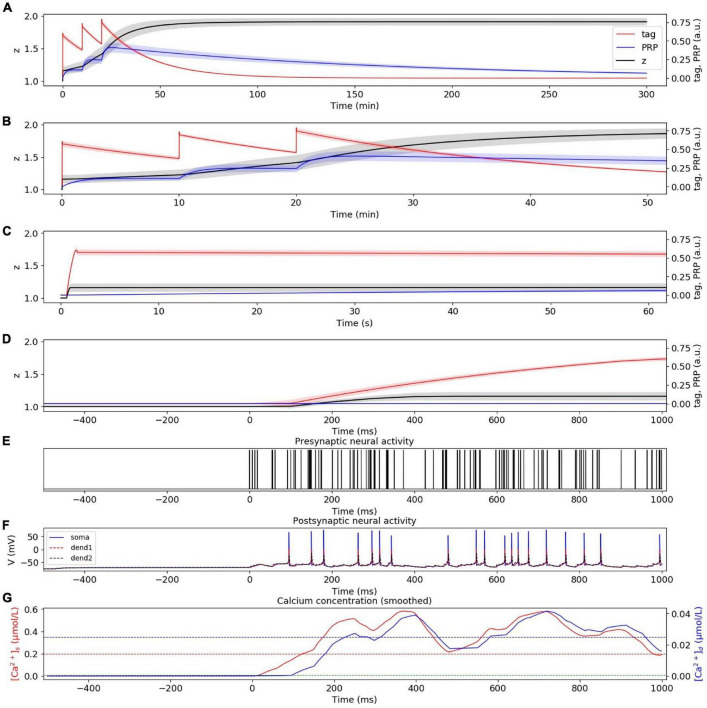
L-LTP induced by strong HFS without considering presynaptic plasticity. **(A)** Dynamic change of tag (red), PRP (blue), and z (black) during the whole simulation time (300 min). **(B)** Dynamic change of tag (red), PRP (blue), and z (black) during the first 50 min. **(C)** Dynamic change of tag (red), PRP (blue), and z (black) during the first 60 s. **(D)** Dynamic change of tag (red), PRP (blue), and z (black) during the first 1 s. **(E)** Activity of presynaptic neuron during the first tetanus stimulus. **(F)** Activity of postsynaptic CA1 neuron during the first tetanus stimulus; the membrane potential of soma, dendrite 1, and dendrite 2 are shown in blue, red, and black. **(G)** Calcium concentration in the spine (red) and dendritic branch (blue) during the first tetanus stimulus; calcium threshold for tag and PRP are shown in green (*Ca*0_*s*_), red (*Ca*1_*s*_), and blue (*Ca*0_*d*_) dotted lines. The PRP has been magnified 200 times for clearer display, and the shaded areas stand for standard deviation.

In the strong HFS experiment without considering presynaptic plasticity, the dynamic changes of tag, PRP, and z under strong HFS were calculated, as shown in Figure 5A. The stimulation at the initial time caused the changes of tag and PRP; thus, we zoom in to visualize the changes of tag, PRP, and z in the first 50 min (Figure 5B), 60 s (Figure 5C), and 1 s (Figure 5D). To visualize the neural activity and calcium concentration behind tag and PRP synthesis, the presynaptic neural activity, postsynaptic neural activity, and calcium concentration in both spine and dendritic branch during the first tetanus stimulus are shown in Figures 5E–G. The calcium concentration in the spine (Figure 5G) is higher than *Ca*1_*s*_ (red dotted line), which is high enough to induce LTP tag. Moreover, the calcium concentration in the dendritic branch is higher than *Ca*0_*d*_ (blue dotted line), which is high enough for PRP synthesis, and the synapse enters L-LTP. During the whole simulation of 5 h, the potentiation of synaptic strength maintains, and the trend of synaptic weight is consistent with observations of the physiological experiment in Figure 2B of Frey and Morris (1997). Moreover, the addition of presynaptic plasticity did not change the tendency of tag, PRP, and z, as shown in Supplementary Figure 2.

#### Strong Low-Frequency Stimuli Induce Late-Phase LTD

**FIGURE 6:**
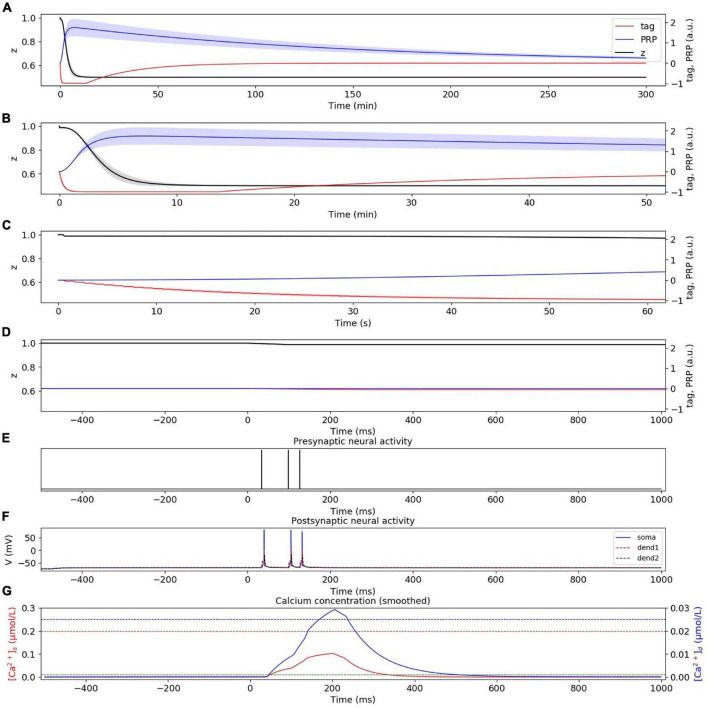
L-LTD induced by strong LFS without considering presynaptic plasticity. **(A)** Dynamic change of tag (red), PRP (blue), and z (black) during the whole simulation time (300 min). **(B)** Dynamic change of tag (red), PRP (blue), and z (black) during the first 50 min. **(C)** Dynamic change of tag (red), PRP (blue), and z (black) during the first 60 s. **(D)** Dynamic change of tag (red), PRP (blue), and z (black) during the first 1 s. **(E)** Activity of presynaptic neuron during the first burst stimulus. **(F)** Activity of postsynaptic CA1 neuron during the first burst stimulus; the membrane potential of soma, dendrite 1, and dendrite 2 are shown in blue, red, and black. **(G)** Calcium concentration in the spine (red) and dendritic branch (blue) during the first burst stimulus, calcium threshold for tag and PRP are shown in green (*Ca*0_*s*_), red (*Ca*1_*s*_), and blue (*Ca*0_*d*_) dotted lines. The PRP has been magnified 200 times for clearer display, and the shaded areas stand for standard deviation.

In the strong LFS experiment without considering presynaptic plasticity, the dynamic changes of tag, PRP, and z under strong LFS are calculated, as shown in Figure 6A. The stimulation at the initial time caused the changes of tag and PRP; thus, we zoom in to visualize the changes of tag, PRP, and z in the first 50 min (Figure 6B), 60 s (Figure 6C), and 1 s (Figure 6D). To visualize the neural activity and calcium concentration behind tag and PRP synthesis, the presynaptic neural activity, postsynaptic neural activity, and calcium concentration in both spine and dendritic branch during the first burst stimulus are shown in Figures 6E–G. The calcium concentration in the spine (Figure 6G) is between *Ca*0_*s*_ (green dotted line) and *Ca*1_*s*_ (red dotted line), which induces LTD tag. The calcium concentration in the dendritic branch is higher than *Ca*0_*d*_ (blue dotted line), which is high enough for PRP synthesis, and the synapse enters L-LTD. During the whole simulation of 5 h, the depression of synaptic strength maintains. The simulation result is consistent with recordings in the physiological experiment in Figure 1B of Sajikumar and Frey (2004). Moreover, the addition of presynaptic plasticity did not change the tendency of tag, PRP, and z, as shown in Supplementary Figure 3.

### Two-Pathway Experiment

#### Strong High-Frequency Stimuli in the First Pathway Induced Plasticity-Related Product Synthesis; Weak High-Frequency Stimuli in the Second Pathway Occurred 30 Min Later Captures Plasticity-Related Product and Enters Late-Phase LTP

**FIGURE 7:**
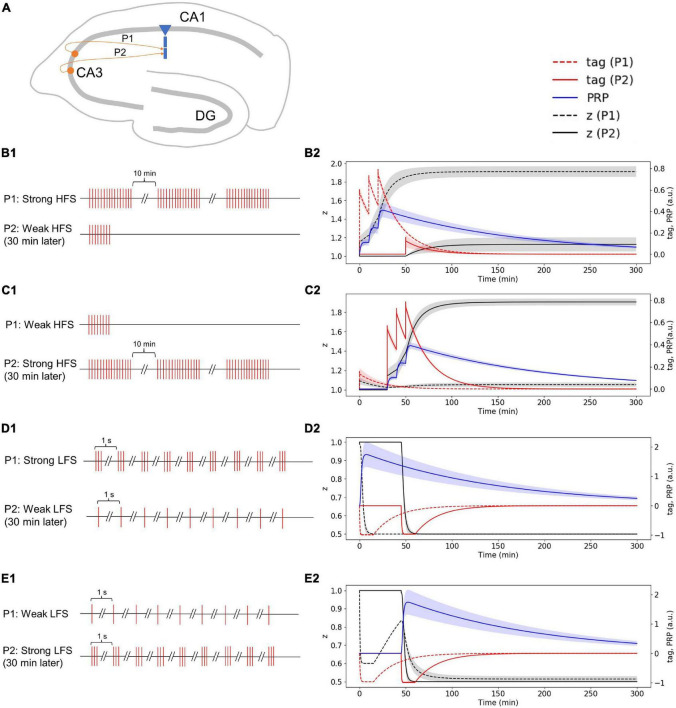
Stimuli and dynamic change of tag, PRP, and z in the two-pathway experiment without considering presynaptic plasticity; both stimuli are high or low frequency. **(A)** Two presynaptic neurons project to the CA1 pyramidal cell in the hippocampus via Schaffer collaterals, the synapse from the first presynaptic neuron is labeled as P1, and the synapse from the second presynaptic neuron is labeled as P2. **(B1)** The stimulus in P1 is strong HFS, and the stimulus in P2 is weak HFS that occurred 30 min later. **(B2)** Strong HFS in P1 induced PRP synthesis; weak HFS in P2 occurred 30 min later captures PRP and enters L-LTP. **(C1)** The stimulus in P1 is weak HFS, and the stimulus in P2 is strong HFS that occurred 30 min later. **(C2)** Weak HFS in P1 induced E-LTP, whereas the strong HFS in P2 occurred 30 min later make the E-LTP convert to L-LTP. **(D1)** The stimulus in P1 is strong LFS, and the stimulus in P2 is weak LFS that occurred 30 min later. **(D2)** Strong LFS in P1 induced PRP synthesis; weak LFS in P2 occurred 30 min later captures PRP and enters L-LTD. **(E1)** The stimulus in P1 is weak LFS, and the stimulus in P2 is strong LFS that occurred 30 min later. **(E2)** Weak LFS in P1 induced E-LTD, whereas the strong LFS in P2 occurred 30 min later make the E-LTD convert to L-LTD. The PRP has been magnified 200 times for clearer display. The shaded areas stand for standard deviation.

In the two-pathway experiment, synapses from different neurons project to one CA1 pyramidal cell through Schaffer collaterals (Figure 7A). The stimulus in the first pathway (P1) is strong HFS, and the stimulus in the second pathway (P2) is weak HFS that occurred 30 min later (Figure 7B1). Without considering presynaptic plasticity, the LTP tag induced by P1 strong HFS is shown in Figure 7B2 (red dotted line), and the stimulus of P1 is strong enough to induce PRP synthesis (blue line in Figure 7B2); thus, the LTP tag captures PRP and enters L-LTP (black dotted line in Figure 7B2). The P2 weak HFS is given 30 min after stimulation of P1 and tags the synapse with LTP (red solid line in Figure 7B2); however, the stimulus of P2 is relatively weak, and it cannot induce the synthesis of PRP. Nevertheless, it captures the PRP synthesized by P1 and enters L-LTP (black solid line in Figure 7B2). During the whole simulation of 5 h, the potentiation of synaptic strength in both P1 and P2 is maintained. The simulation result is consistent with observations in the physiological experiment in Figure 4D of Frey and Morris (1997). Moreover, the addition of presynaptic plasticity did not change the tendency of tag, PRP, and z, and we found the increase of L-LTP amplitude of P2, as shown in Supplementary Figure 4A.

#### Weak High-Frequency Stimuli in the First Pathway Induced Early Phase LTP, Whereas the Strong High-Frequency Stimuli in the Second Pathway Occurred 30 Min Later Make the Early Phase LTP Convert to Late-Phase LTP

The stimulus in P1 is weak HFS, and the stimulus in P2 is strong HFS that occurred 30 min later (Figure 7C1). Without considering presynaptic plasticity, the LTP tag induced by P1 weak HFS is shown in Figure 7C2 (red dotted line); however, the stimulus of P1 is too weak to induce PRP synthesis, thus, the synapse in P1 enters E-LTP (black dotted line in Figure 7C2). P2 strong HFS is given 30 min after stimulation of P1 and tags the synapse with LTP (red solid line in Figure 7C2), and the stimulus of P2 is strong enough to induce the synthesis of PRP, P2 enters L-LTP directly (black solid line in Figure 7C2). Then, the LTP tag of P1 captures the PRP synthesized by P2 and converts E-LTP to L-LTP (black dotted line in Figure 7C2). During the whole simulation of 5 h, the potentiation of synaptic strength in both P1 and P2 is maintained. The simulation result is consistent with recordings of the physiological experiment in Figure 2D of Frey and Morris (1998). Moreover, the addition of presynaptic plasticity did not change the tendency of tag, PRP, and z, and we found the increase of L-LTP amplitude of P1, as shown in Supplementary Figure 4B.

#### Strong Low-Frequency Stimuli in the First Pathway Induced Plasticity-Related Product Synthesis; Weak Low-Frequency Stimuli in the Second Pathway Occurred 30 Min Later Captures Plasticity-Related Product and Enters Late-Phase LTD

The stimulus in P1 is strong LFS, and the stimulus in P2 is weak LFS that occurred 30 min later (Figure 7D1). Without considering presynaptic plasticity, the LTD tag induced by P1 strong LFS is shown in Figure 7D2 (red dotted line), and the stimulus of P1 is strong enough to induce PRP synthesis (blue line in Figure 7D2); thus, the LTD tag captures the PRP and enters L-LTD directly (black dotted line in Figure 7D2). P2 weak LFS is given 30 min after stimulation of P1 and tags the synapse with LTD (red solid line in Figure 7D2); however, the stimulus of P2 is relatively weak, and it cannot induce the synthesis of PRP. Nevertheless, it captures the PRP synthesized by P1 and enters L-LTD (black solid line in Figure 7D2). During the whole simulation of 5 h, the synaptic depression of P1 and the synaptic potentiation of P2 is maintained. The simulation result is consistent with recordings in the physiological experiment in Figure 2B of Sajikumar and Frey (2004). Moreover, the addition of presynaptic plasticity did not change the tendency of tag, PRP, and z, as shown in Supplementary Figure 4C.

#### Weak Low-Frequency Stimuli in the First Pathway Induced Early Phase LTD, Whereas the Strong Low-Frequency Stimuli in the Second Pathway Occurred 30 Min Later Make the Early Phase LTD Convert to Late-Phase LTD

The stimulus in P1 is weak LFS, and the stimulus in P2 is strong LFS that occurred 30 min later (Figure 7E1). Without considering presynaptic plasticity, the LTD tag induced by P1 weak LFS is shown in Figure 7E2 (red dotted line); however, the stimulus of P1 is too weak to induce PRP synthesis, and thus, the synapse in P1 enters E-LTD (black dotted line in Figure 7E2). P2 strong LFS is given 30 min after stimulation of P1 and tags the synapse with LTD (red solid line in Figure 7E2), and the stimulus of P2 is strong enough to induce the synthesis of PRP; P2 enters L-LTD directly (black solid line in Figure 7E2). Then, the LTD tag of P1 captures the PRP synthesized by P2 and converts E-LTD to L-LTD (black dotted line in Figure 7E2). During the whole simulation of 5 hours, the synaptic depression of P1 and the synaptic potentiation of P2 is maintained. The simulation result is consistent with observations in the physiological experiment in Figure 2C of Sajikumar and Frey (2004). Moreover, the addition of presynaptic plasticity did not change the tendency of tag, PRP, and z, as shown in Supplementary Figure 4D.

#### Strong High-Frequency Stimuli in the First Pathway Induced Plasticity-Related Product Synthesis; Weak Low-Frequency Stimuli in the Second Pathway Occurred 30 Min Later Captures Plasticity-Related Product, and Enters Late-Phase LTD

**FIGURE 8:**
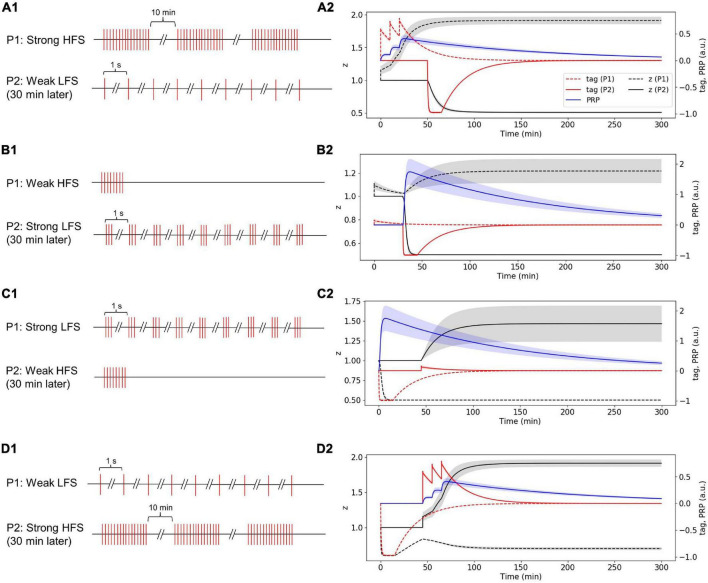
Stimuli and dynamic change of tag, PRP, and z in the two-pathway experiment without considering presynaptic plasticity; one stimulus is high frequency, and the other stimulus is low frequency. **(A1)** The stimulus in P1 is strong HFS, and the stimulus in P2 is weak LFS that occurred 30 min later. **(A2)** Strong HFS in P1 induced PRP synthesis; weak LFS in P2 occurred 30 min later and captures PRP and enters L-LTD. **(B1)** The stimulus in P1 is weak HFS, and the stimulus in P2 is strong LFS that occurred 30 min later. **(B2)** Weak HFS in the first pathway induced E-LTP, whereas the strong LFS in the second pathway occurred 30 min later to make the E-LTP convert to L-LTP. **(C1)** The stimulus in P1 is strong LFS, and the stimulus in P2 is weak HFS that occurred 30 min later. **(C2)** Strong LFS in the first pathway induced PRP synthesis; weak HFS in the second pathway occurred 30 min later and captures PRP and enters L-LTP. **(D1)** The stimulus in P1 is weak LFS, and the stimulus in P2 is strong HFS that occurred 30 min later. **(D2)** Weak LFS in the first pathway induced E-LTD, whereas the strong HFS in the second pathway occurred 30 min later to make the E-LTD convert to L-LTD. The PRP has been magnified 200 times for clearer display. The shaded areas stand for standard deviation.

The stimulus in P1 is strong HFS, and the stimulus in P2 is weak LFS that occurred 30 min later (Figure 8A1). Without considering presynaptic plasticity, the LTP tag induced by P1 strong HFS is shown in Figure 8A2 (red dotted line), and the stimulus of P1 is strong enough to induce PRP synthesis (blue line in Figure 8A2); thus, the LTP tag captures the PRP and enters L-LTP directly (black dotted line in Figure 8A2). P2 weak LFS is given 30 min after stimulation of P1 and tags the synapse with LTD (red solid line in Figure 8A2); however, the stimulus of P2 is relatively weak, and it cannot induce the synthesis of PRP. Nevertheless, it captures the PRP synthesized by P1 and enters L-LTD (black solid line in Figure 8A2). During the whole simulation of 5 h, the synaptic potentiation of P1 and synaptic depression of P2 is maintained. The simulation result is consistent with observations in the physiological experiment in Figure 4D of Sajikumar and Frey (2004). Moreover, the addition of presynaptic plasticity did not change the tendency of tag, PRP, and z, as shown in Supplementary Figure 5A.

#### Weak High-Frequency Stimuli in the First Pathway Induced Early Phase LTP, Whereas the Strong Low-Frequency Stimuli in the Second Pathway Occurred 30 Min Later Make the E-LTP Convert to L-LTP

The stimulus in P1 is weak HFS, and the stimulus in P2 is strong LFS that occurred 30 min later (Figure 8B1). Without considering presynaptic plasticity, the LTP tag induced by P1 weak HFS is shown in Figure 8B2 (red dotted line); however, the stimulus of P1 is too weak to induce PRP synthesis, and thus, the synapse in P1 enters E-LTP (black dotted line in Figure 8B2). P2 strong LFS is given 30 min after stimulation of P1 and tags the synapse with LTD (red solid line in Figure 8B2), and the stimulus of P2 is strong enough to induce the synthesis of PRP; P2 enters L-LTD directly (black solid line in Figure 8B2). Then, the LTP tag of P1 captures the PRP synthesized by P2 and converts E-LTP to L-LTP (black dotted line in Figure 8B2). During the whole simulation of 5 h, the synaptic potentiation of P1 and synaptic depression of P2 is maintained. The simulation result is consistent with observations in the physiological experiment in Figure 4A of Sajikumar and Frey (2004). Moreover, the addition of presynaptic plasticity did not change the tendency of tag, PRP, and z, and we found the increase of L-LTP amplitude of P1, as shown in Supplementary Figure 5B.

#### Strong Low-Frequency Stimuli in the First Pathway Induced Plasticity-Related Product Synthesis; Weak High-Frequency Stimuli in the Second Pathway Occurred 30 Min Later Captures Plasticity-Related Product and Enters Late-Phase LTP

The stimulus in P1 is strong LFS, and the stimulus in P2 is weak HFS that occurred 30 min later (Figure 8C1). Without considering presynaptic plasticity, the LTD tag induced by P1 strong LFS is shown in Figure 8C2 (red dotted line), and the stimulus of P1 is strong enough to induce PRP synthesis (blue line in Figure 8C2); thus, the LTD tag captures the PRP and enters L-LTD directly (black dotted line in Figure 8C2). P2 weak HFS is given 30 min after stimulation of P1 and tags the synapse with LTP (red solid line in Figure 8C2); however, the stimulus of P2 is relatively weak, and it cannot induce the synthesis of PRP. Nevertheless, it captures the PRP synthesized by P1 and enters L-LTP (black solid line in Figure 8C2). During the whole simulation of 5 h, the synaptic depression of P1 and synaptic potentiation of P2 is maintained. The simulation result is consistent with observations in the physiological experiment in Figure 4B of Sajikumar and Frey (2004). Moreover, the addition of presynaptic plasticity did not change the tendency of tag, PRP, and z, and we found the increase of L-LTP amplitude of P2, as shown in Supplementary Figure 5C.

#### Weak Low-Frequency Stimuli in the First Pathway Induced Early Phase LTD, Whereas the Strong High-Frequency Stimuli in the Second Pathway Occurred 30 Min Later Make the Early Phase LTD Convert to Late-Phase LTD

The stimulus in P1 is weak LFS, and the stimulus in P2 is strong HFS that occurred 30 min later (Figure 8D1). Without considering presynaptic plasticity, the LTD tag induced by P1 weak LFS is shown in Figure 8D2 (red dotted line); however, the stimulus of P1 is too weak to induce PRP synthesis, and thus, the synapse in P1 enters E-LTD (black dotted line in Figure 8D2). P2 strong HFS is given 30 min after stimulation of P1 and tags the synapse with LTP (red solid line in Figure 8D2), and the stimulus of P2 is strong enough to induce the synthesis of PRP; P2 enters L-LTP directly (black solid line in Figure 8D2). Then, the LTD tag of P1 captures the PRP synthesized by P2 and converts E-LTD to L-LTD (black dotted line in Figure 8D2). During the whole simulation of 5 h, the synaptic depression of P1 and the synaptic potentiation of P2 is maintained. The simulation result is consistent with observations in the physiological experiment in Figure 4C of Sajikumar and Frey (2004). Moreover, the addition of presynaptic plasticity did not change the tendency of tag, PRP, and z, as shown in Supplementary Figure 5D.

## Discussion

In this study, according to the STC theory, we propose the SM-STC model and simulate various plasticity phenomena on Schaffer collateral synaptic connections to the CA1 pyramidal neuron. The simulation results in both single- and two-pathway experiments are consistent with physiological observations (Frey and Morris, 1997, 1998; Sajikumar and Frey, 2004). In the single-pathway experiments, weak HFS/LFS is used to induce E-LTP/LTD, which could sustain for about 90 min (Figures 4, 5); strong HFS/LFS is used to induce L-LTP/LTD, which are well-maintained for the 5 h of biological time we simulated (Figures 6, 7), suggesting that, with longer simulation time, L-LTP and L-LTD can still maintain. In the two-pathway experiments, the weak stimuli from one pathway could tag the synapse and trigger E-LTP/D, but they are not strong enough to induce PRP synthesis in the dendritic branch; thus the E-LTP/D could not convert to L-LTP/D. However, if strong stimuli in another pathway leading to the synthesis of PRP in the same dendritic branch and the PRP arrives prior to the decay of the tag, the E-LTP/D could be transformed into L-LTP/D, which is known as cross-capture (Figures 7, 8). Furthermore, we simulated the synaptic change by considering both presynaptic and postsynaptic plasticity. The simulation results show that after the addition of presynaptic plasticity, the tendency of tag, PRP and z does not change, and the amplitude of LTP in some simulations increased, which fills the gap of the physiological experiment that is hard to include presynaptic plasticity *in vitro*. Therefore, the proposed SM-STC model combines presynaptic efficiency (facilitation and depression) and postsynaptic plasticity (potentiation and depression), which brought us a more complete map of brain-like dynamics. Presynaptic efficiency changes in a fast time scale, whereas postsynaptic plasticity based on synaptic tagging and PRP capture is in a slow time scale; these diverse synaptic plasticity mechanisms could orchestrate for more biological plausible simulations (Zenke et al., 2015).

Compared with previous models, the SM-STC model in this study considers biochemical mechanisms and implements simplicity simultaneously, which could bridge the gap between STC theory and behavior. Building spiking neural networks with SM-STC as a learning rule could help us understand the neural basis of associative memory. Memory is deemed to be represented in the form of cell assembly (or engram) (Buzsáki, 2010; Josselyn et al., 2015), and related memories are suggested to share neurons for association (De Falco et al., 2016). However, whether these associated memories shared the same (clustered distribution) or a different dendritic branch (dispersed distribution) matters in the relationship between them. For example, if two memories share the same dendritic branch, the enhancement or degradation of one memory will cause the enhancement or decline of another memory; however, if two memories share different dendritic branches of the same neuron, the enhancement or degradation of one memory may not affect the other memory. In addition, due to the locality of PRP, memories experienced close in time are more likely to be encoded in the same dendritic branch (Cai et al., 2016; Kastellakis et al., 2016); thus, SM-STC provides a good starting point for neural network modeling on memory presentation on the synaptic level.

The SM-STC model could also be used in studying reinforcement learning. The dopamine release during a novel experience is crucial for the establishment of novelty-induced memory (Okuda et al., 2021). Activation of dopamine D1/D5 receptors causes increased availability of PRPs (Frey and Morris, 1998; Redondo and Morris, 2011), whereas inhibition of dopamine D1/D5 receptors prevents the STC process by blocking the synthesis of PRP (Wang et al., 2010). In addition, experiments on both rodents (Morris, 2006; Moncada and Viola, 2007) and humans (Ramirez Butavand et al., 2020) show that experiencing unexpected novelty before or after learning could enhance memory. Thus, adding dopamine neurons in the spiking neural network with SM-STC could shed light on reinforcement learning tasks.

The presented SM-STC model can also help understand memory decline in aged individuals (Shetty and Sajikumar, 2017). In aged rodents, the E-LTP induced by weak stimuli could not convert to L-LTP, whereas strong stimuli could induce L-LTP with the potentiation amplitude lower than young rodents (Sharma et al., 2015; Shetty et al., 2017), which may be caused by unstable tag or decreased PRP synthesis during weak stimuli. These influence factors can be reflected by changing parameters in the SM-STC model, which provides the possibility for understanding memory decline in aged individuals by neural network modeling.

The performance of the SM-STC model has been tested in a neuron model with three compartments. The SM-STC model can also be used in neurons with biological morphology, which have hundreds or thousands of compartments. A single-neuron model with biological morphology is shown to have strong computational power (Gidon et al., 2020; Beniaguev et al., 2021). Moreover, single neurons can learn network-level computations simply by tuning synaptic weights (Bicknell and Häusser, 2021). Thus, the SM-STC model could provide a new, simple, general, and biologically reasonable learning rule for neural networks with complex morphological neurons.

The SM-STC model depicts transmitter and residual calcium level–induced plasticity based on presynaptic neural activity and early/late-phase long-term plasticity dependent on postsynaptic activity on excitatory synapses. Because plasticity manifests in multiple concurrently active forms in the brain (Citri and Malenka, 2008), the SM-STC model cannot capture all the circumstances. In terms of time scale, the plasticity rule could be divided into rapidly induced plasticity lasting a few seconds to tens of seconds (Zucker and Regehr, 2002) and homeostatic plasticity lasting for hours or even longer (Turrigiano and Nelson, 2000). From the viewpoint of spatial scale, the plasticity rule includes local plasticity depending only on the activity of the presynaptic and postsynaptic neuron and global plasticity modulated by global factors, such as neuromodulator (Turrigiano, 2012). According to the neurotransmitter types of presynaptic neurons, the plasticity could be divided into excitatory and inhibitory plasticity (Kullmann et al., 2012; Froemke, 2015). Therefore, the SM-STC model provides a biologically plausible rule for rapidly induced, homeostatic, and local plasticity and could work together with global and inhibitory plasticity to bring a complete map of neural plasticity.

In summary, we propose a synaptic plasticity model SM-STC that takes biological rationality and simplicity into account simultaneously, and we demonstrate the effectiveness of the model by a series of simulation experiments. The SM-STC model could bridge the gap between STC theory and behavior performance and provide new insight for modeling memory association, reinforcement learning, and memory decline in aged adults through neural networks.

## Data Availability Statement

The original contributions presented in the study are included in the article/Supplementary Material, further inquiries can be directed to the corresponding author/s.

## Author Contributions

YW and LC proposed the model and designed the experiments. YD carried out the experiments and performed the data analyses. YD and YW wrote the first draft of the manuscript. All authors contributed to manuscript revision, read, and approved the submitted version.

## Conflict of Interest

The authors declare that the research was conducted in the absence of any commercial or financial relationships that could be construed as a potential conflict of interest.

## Publisher’s Note

All claims expressed in this article are solely those of the authors and do not necessarily represent those of their affiliated organizations, or those of the publisher, the editors and the reviewers. Any product that may be evaluated in this article, or claim that may be made by its manufacturer, is not guaranteed or endorsed by the publisher.
