# Supplementary material for: A Simplified Plasticity Model Based on Synaptic Tagging and Capture Theory: Simplified STC
Source: Front Comput Neurosci. 2022 Feb 11;15:798418. doi: 10.3389/fncom.2021.798418 (PMC8873158; doi:10.3389/fncom.2021.798418)
Supplement: Supplementary file 1 [file Image_1.PDF]

Supplementary Figures

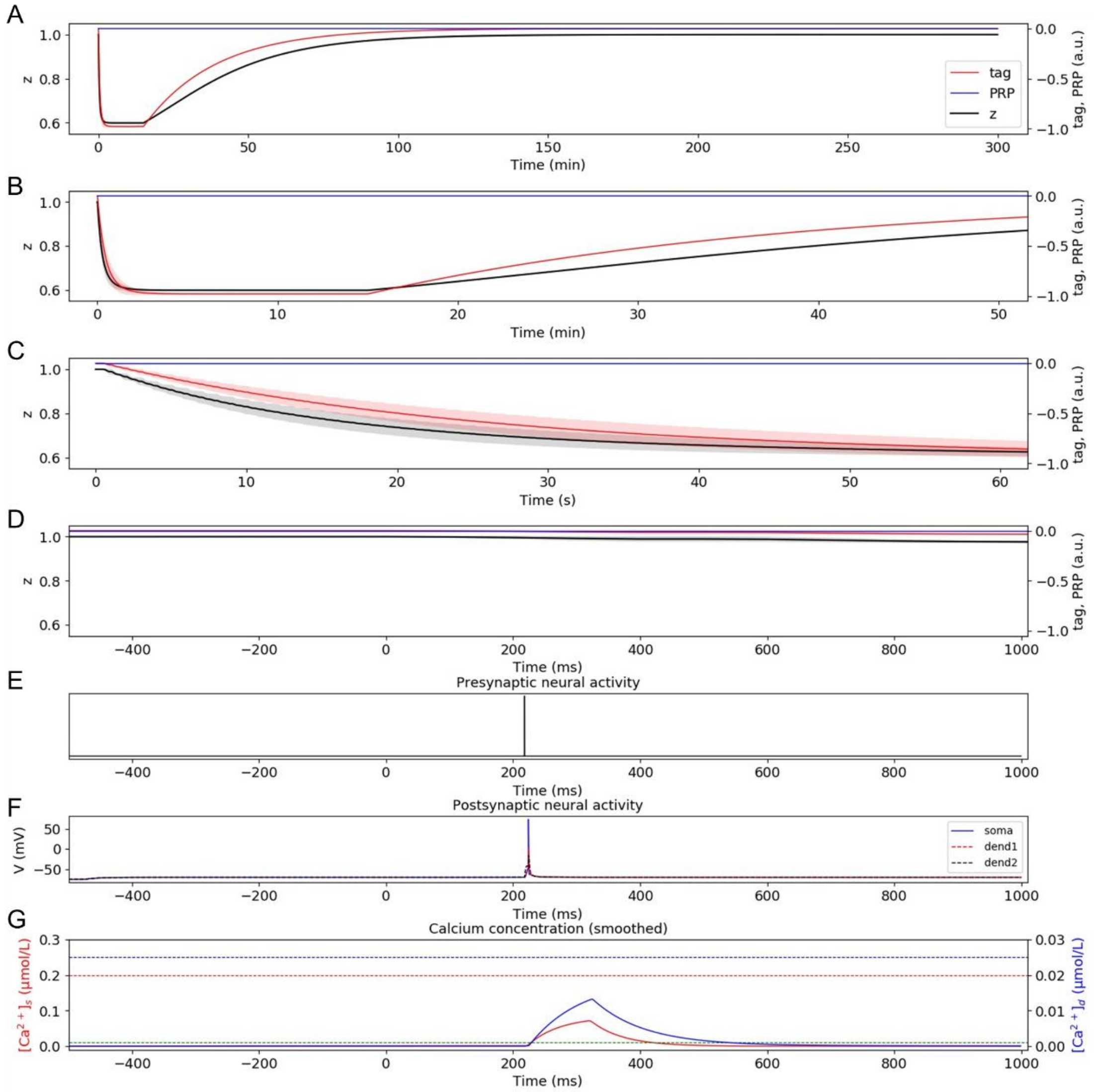

Figure S1. E-LTD induced by weak LFS considering presynaptic plasticity. (A) Dynamic change of tag (red), PRP (blue) and z (black) during the whole simulation time (300 min); (B) Dynamic change of tag (red), PRP (blue) and z (black) during the first 50 min; (C) Dynamic change of tag (red), PRP (blue) and z (black) during the first 60 s; (D) Dynamic change of tag (red), PRP (blue) and z (black) during the first 1 s; (E) Activity of presynaptic neuron during the first 1 Hz stimulus; (F) Activity of postsynaptic CA1 neuron, the membrane potential of soma, dendrite 1, and dendrite 2 are shown in blue, red, and black; (G) Calcium concentration in spine (red) and dendritic branch (blue), calcium threshold for tag and PRP are shown in green ($Ca0_s$), red ($Ca1_s$) and blue ($Ca0_d$) dotted line. The PRP has been magnified 200 times for clearer display, and the shaded areas stand for standard deviation.

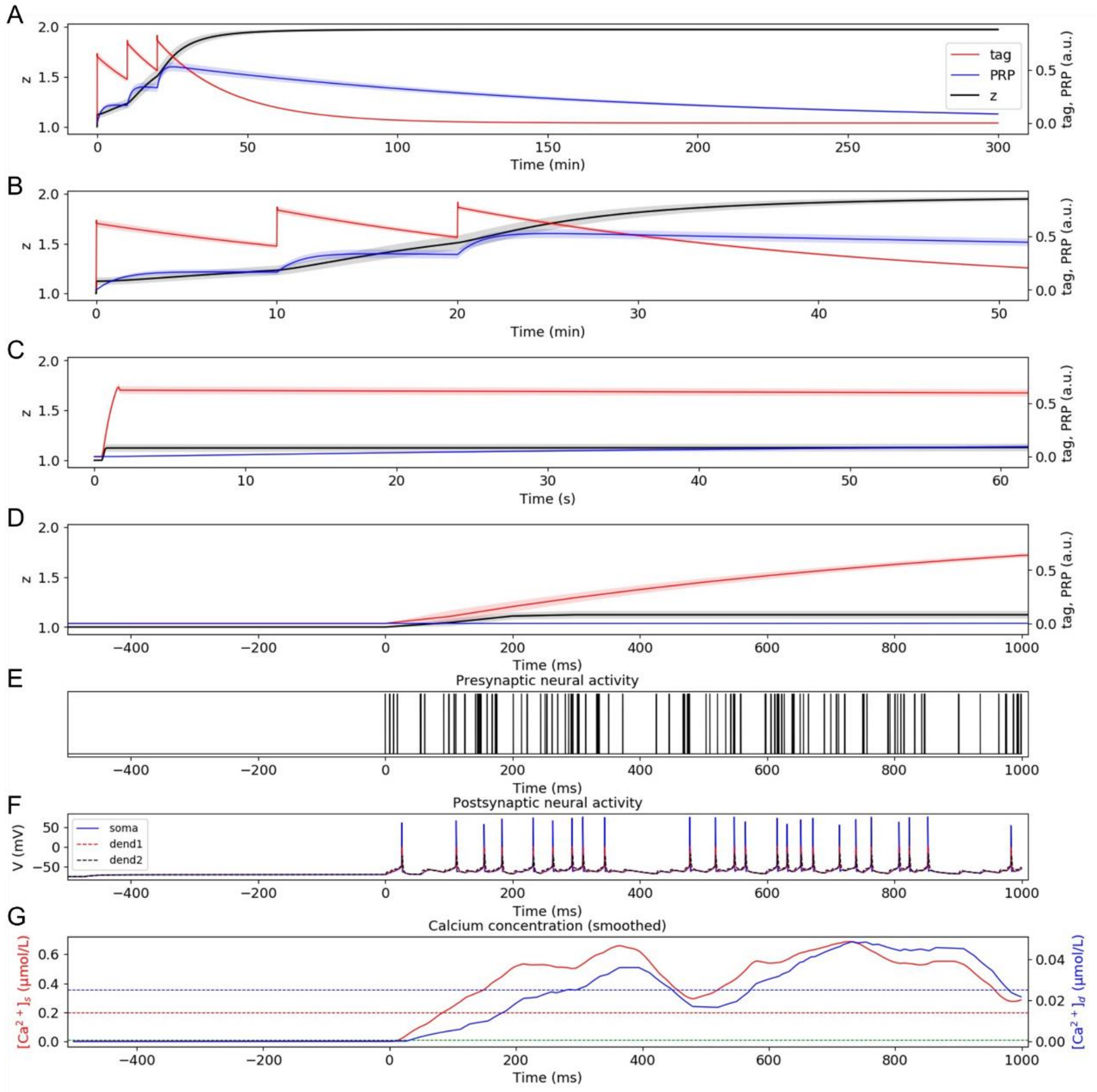

Figure S2. L-LTP induced by strong HFS considering presynaptic plasticity. (A) Dynamic change of tag (red), PRP (blue) and z (black) during the whole simulation time (300 min); (B) Dynamic change of tag (red), PRP (blue) and z (black) during the first 50 min; (C) Dynamic change of tag (red), PRP (blue) and z (black) during the first 60 s; (D) Dynamic change of tag (red), PRP (blue) and z (black) during the first 1 s; (E) Activity of presynaptic neuron during the first tetanus stimulus; (F) Activity of postsynaptic CA1 neuron during the first tetanus stimulus, the membrane potential of soma, dendrite 1, and dendrite 2 are shown in blue, red, and black; (G) Calcium concentration in spine (red) and dendritic branch (blue) during the first tetanus stimulus, calcium threshold for tag and PRP are shown in green ($Ca0_s$), red ($Ca1_s$) and blue ($Ca0_d$) dotted line. The PRP has been magnified 200 times for clearer display, and the shaded areas stand for standard deviation.

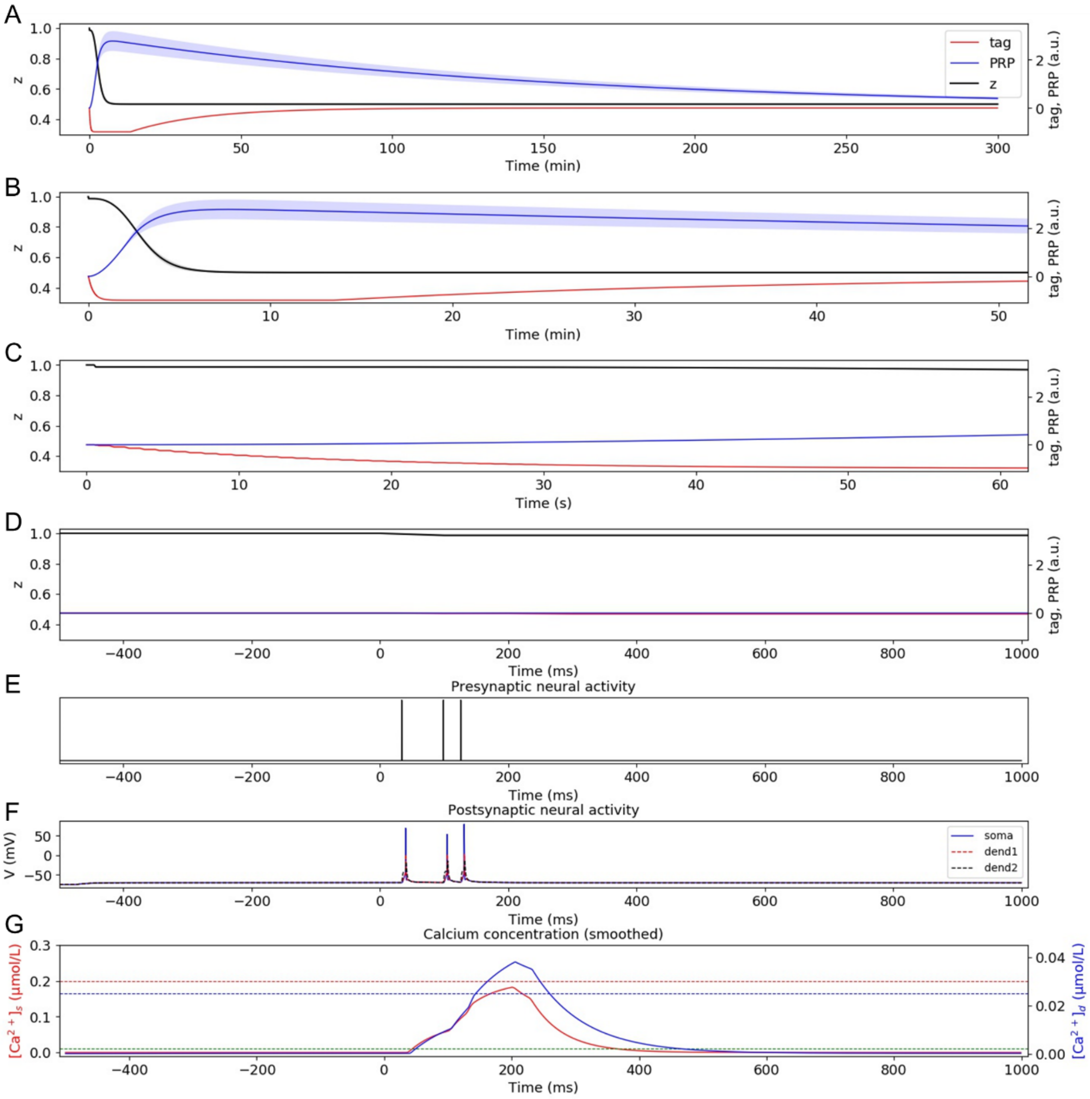

Figure S3. L-LTD induced by strong LFS considering presynaptic plasticity. (A) Dynamic change of tag (red), PRP (blue) and z (black) during the whole simulation time (300 min); (B) Dynamic change of tag (red), PRP (blue) and z (black) during the first 50 min; (C) Dynamic change of tag (red), PRP (blue) and z (black) during the first 60 s; (D) Dynamic change of tag (red), PRP (blue) and z (black) during the first 1 s; (E) Activity of presynaptic neuron during the first burst stimulus; (F) Activity of postsynaptic CA1 neuron during the first burst stimulus, the membrane potential of soma, dendrite 1, and dendrite 2 are shown in blue, red, and black; (G) Calcium concentration in spine (red) and dendritic branch (blue) during the first burst stimulus, calcium threshold for tag and PRP are shown in green ($Ca0_s$), red ($Ca1_s$) and blue ($Ca0_d$) dotted line. The PRP has been magnified 200 times for clearer display, and the shaded areas stand for standard deviation.

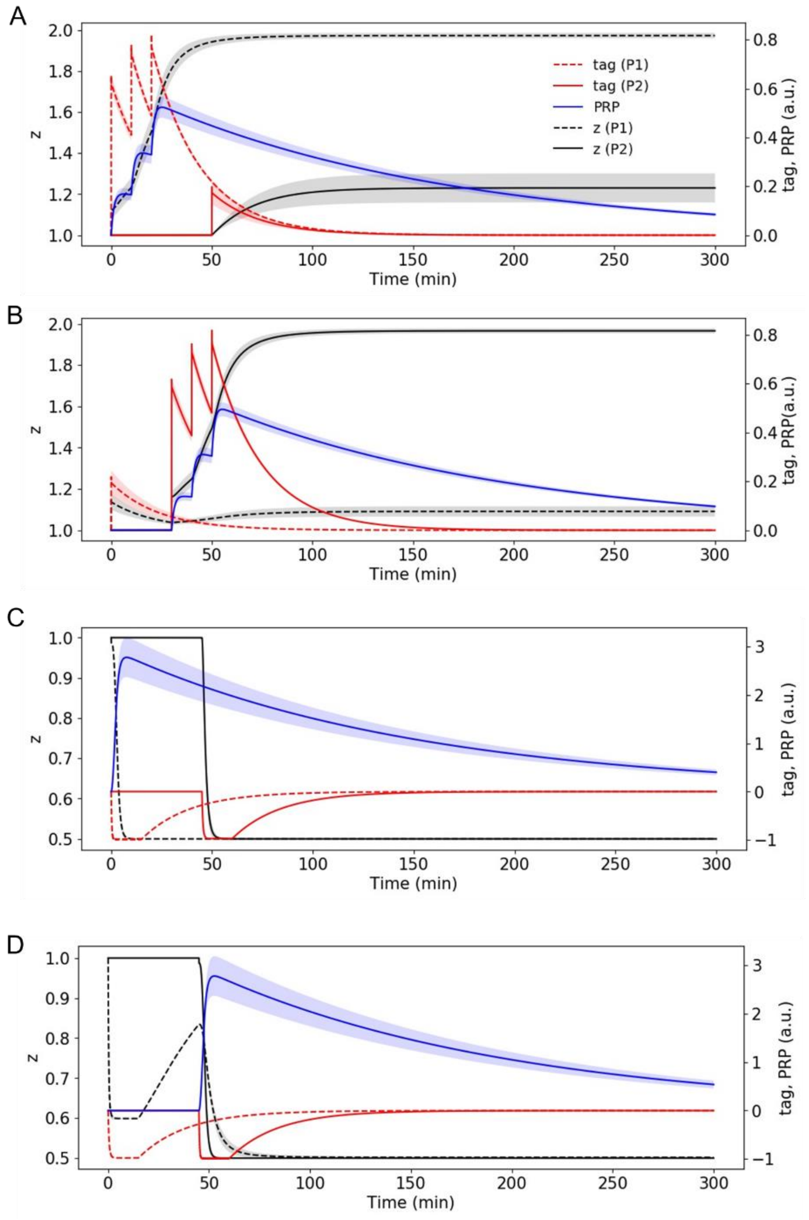

Figure S4. Dynamic change of tag, PRP, and z in two-pathway experiment considering presynaptic plasticity, both stimuli are high frequency or low frequency. (A) Strong HFS in P1 induced PRP synthesis, weak HFS in P2 occurred 30 min later captures PRP and enters L-LTP. (B) Weak HFS in P1 induced E-LTP, while the strong HFS in P2 occurred 30 min later make the E-LTP convert to L-LTP. (C) Strong LFS in P1 induced PRP synthesis, weak LFS in P2 occurred 30 min later captures PRP and enters L-LTD. (D) Weak LFS in P1 induced E-LTD, while the strong LFS in P2 occurred 30 min later make the E-LTD convert to L-LTD. The PRP has been magnified 200 times for clearer display. The shaded areas stand for standard deviation.

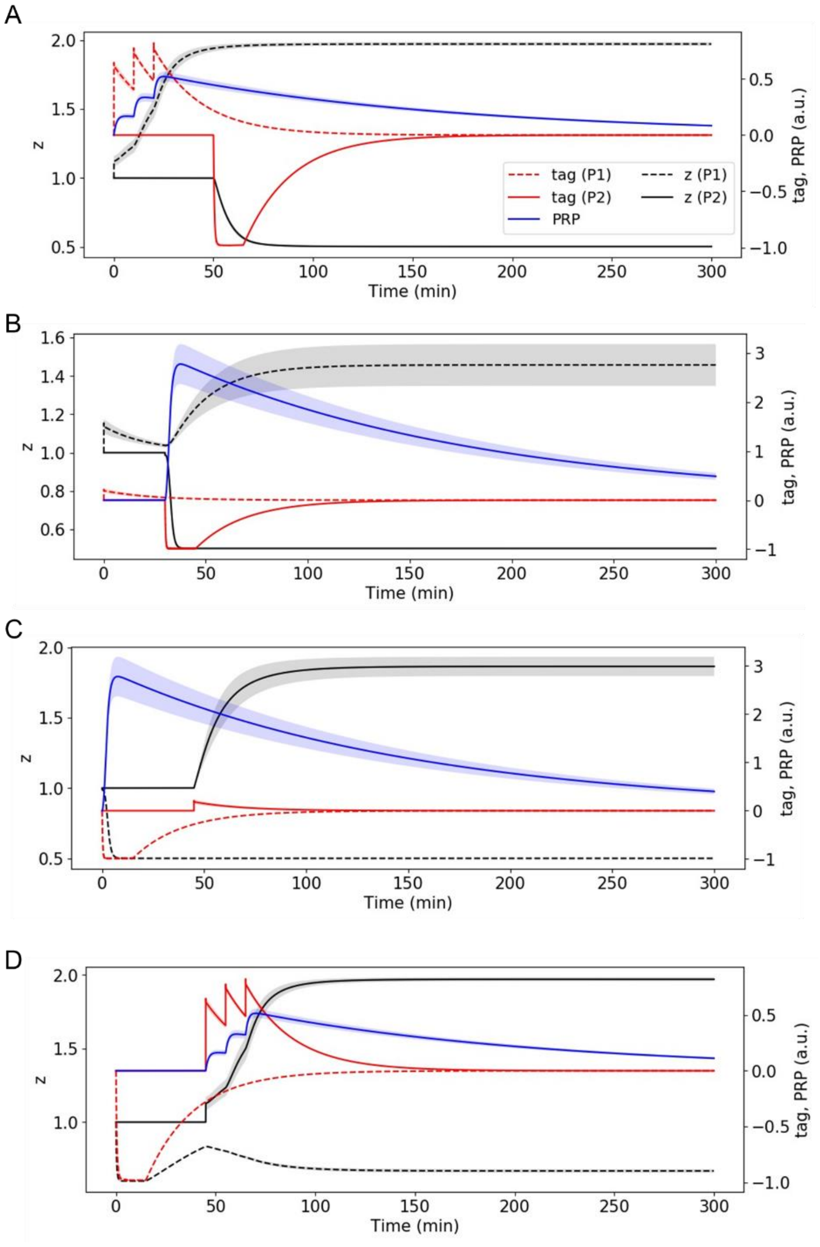

Figure S5. Dynamic change of tag, PRP, and z in two-pathway experiment considering presynaptic plasticity, one stimulus is high frequency, and the other stimulus is low frequency. (A) Strong HFS in P1 induced PRP synthesis, weak LFS in P2 occurred 30 min later captures PRP and enters L-LTD. (B) Weak HFS in the first pathway induced E-LTP, while the strong LFS in the second pathway occurred 30 min later make the E-LTP convert to L-LTP. (C) Strong LFS in the first pathway induced PRP synthesis, weak HFS in the second pathway occurred 30 min later captures PRP and enters L-LTP. (D) Weak LFS in the first pathway induced E-LTD, while the strong HFS in the second pathway occurred 30 min later make the E-LTD convert to L-LTD. The PRP has been magnified 200 times for clearer display. The shaded areas stand for standard deviation.
